# Keratometry device for surgical support

**DOI:** 10.1186/1475-925X-8-37

**Published:** 2009-12-08

**Authors:** Liliane Ventura, Jean-Jacques De Groote, Paula Saia, Sidney J Faria e Sousa

**Affiliations:** 1 Dept of Elect Engineering USP (Ophthalmic Instrumentation Division), Av Trabalhador sao-carlense 400, S Carlos, SP 13566-590, Brasil; 2 Dept of Ophthalmology USP, Av Bandeirantes 3900, Rib Preto, SP 14100-900, Brasil

## Abstract

**Background:**

High astigmatisms are usually induced during corneal suturing subsequent to tissue transplantation or any other surgery which involves corneal suturing. One of the reasons is that the procedure is intimately dependent on the surgeon's skill for suturing identical stitches. In order to evaluate the influence of the irregularity on suturing for the residual astigmatism, a prototype for ophthalmic surgical support has been developed. The final intention of this prototype is to be an evaluation tool for guided suture and as an outcome diminish the postoperative astigmatism.

**Methods:**

The system consists of hand held ring with 36 infrared LEDs, that is to be projected onto the lachrymal film of the cornea. The image is reflected back through the optics of the ocular microscope and its distortion from the original circular shape is evaluated by developed software. It provides keratometric and circularity measurements during surgery in order to guide the surgeon for uniformity in suturing.

**Results:**

The system is able to provide up to 23D of astigmatism (32D - 55D range) and is ± 0.25D accurate. It has been tested in 14 volunteer patients intraoperative and has been compared to a commercial keratometer Nidek Oculus Hand-held corneal topographer. The correlation factors are 0.92 for the astigmatism and 0.97 for the associated axis.

**Conclusion:**

The system is potentially efficient for guiding the surgeon on uniformity of suturing, presenting preliminary data indicating an important decrease on the residual astigmatism, from an average of 8D - for patients not submitted to the prototype guidance - to 1.4D - for patients who have actually been submitted to the prototype guidance - after the first 24 hours post-surgery and in the subsequent weeks. It also indicates that the surgeon should achieve circularity greater or equal to 98% in order to avoid postoperative astigmatisms over 1D.

**Trial Registration:**

Trial registration number: CAAE - 0212.0.004.000-09.

## Background

One of the significant provoking factors of corneal astigmatism during the surgical procedure is the irregularity in suturing the tissue. Sutures are manually performed on the cornea and systems for monitoring the circularity of the suturing [1-4] are not often used. Most of them are just qualitative.

Refractors [5] are used to measure the astigmatism of the eye, which is a refractive error of the ocular medium. keratometers are instruments used to measure objectively the radii of corneal curvature along the two main meridians of the cornea. They provide the mean corneal astigmatism. Most of the current keratometers perform the measurements on patients' eyes at the sitting up position, i.e. in a vertical position. Only a few commercially available models are apt to perform keratometry on a patient in a lying down position, i.e., horizontally positioned. Therefore, some surgeons consider hand held surgical keratometers unreliable [6] and it may be due to the fact of positioning it, as well as, due to measurements that are performed in horizontal position are, to some extent, different from measurements performed in a vertical position.

The optical principle of keratometers is already well known [7] and some of the current authors have previously developed a keratometer module for slit lamps, which performs keratometry with the patient in the vertical position [8-10].

The intention of the present work is to evaluate the postoperative residual astigmatism to understand if a regular suturing is of any benefit for the patient.

Thus, we have developed a system that allows automated keratometry as well as an objective real-time orientation for the surgeon for suturing the corneal tissue as circular as possible, expecting to reduce the residual the post-operative corneal astigmatism. The goal is to develop a prototype and do preliminary tests on the device and not do a full-blown clinical study to prove its commercial and daily clinical routine availability. It is mostly to check about its influence on guiding suturing as a better result for the postoperative astigmatism.

A brief description of the optical properties of the human cornea that concerns this work will be made as well as the calculus involved for determining the radii of curvature of the cornea will be described.

### Optical Properties of the Human Cornea

The human cornea is the optical component, which is the surface with external contact with the light that penetrates the eye; therefore its shape and physical structure are intimately related to the quality of vision.

The shape of the cornea is not spherical overall, it is actually ellipsoidal, but the 3 mm central region of the cornea [11,12] - the central optical zone - where keratometry is performed, may be considered as spherical. The adult cornea is 11-12 mm wide in the horizontal plane and 9-11 mm in the vertical plane. The circumference of its base has a diameter of approximately 12.5 mm. It is approximately 500 μm thick at the central portion and up to 700 μm thick at the periphery [12-15].

The corneal radius of curvature is not regular in its entire surface; it is smaller at the centre (higher curvature) and flattens towards the periphery, causing an aspherical surface.

The radius of curvature in the central optical zone varies form 7.5 - 8.0 mm (typically 7.8 mm), its refractive index is n = 1.376 and the refractive power of the cornea varies from 40 - 44D (the cornea is responsible for 2/3 of the overall refractive power of the eye).

The cornea is equivalent to a positive lens. However it is a very thin structure and the central part of the cornea may be considered as having parallel faces for keratometry purposes [11,12].

The cornea may be considered as a convex mirror, which provides a virtual image of the projected light source structure.

For keratometry purposes just the central optical zone will be analysed, thus the spherical model is sufficient and accurate, but for small radii of curvature, no approximations should be made, as we have learned from the development and tests of our first keratometry system for slit lamps [8-10].

If an object of size *2R *(*R *is the radius of the circular target) is perfectly focused onto the cornea, the reflection of the object provides a virtual image *i*, which corresponds to one half of the dimension of the projected target onto the cornea (around 1.5 mm) [9].

The distance between the object and its image *d *is 0.20 m for the surgical ocular microscope that we have used.

Considering that the light rays coming from *R *reach the optical axis at small angles, it is possible to say that the focusing distance is one half of the curvature radius of the cornea (*r*). Therefore, the radius of curvature *r *(in meters) of the surface to be determined is [9]:(1)

The keratometric results are usually presented as "Refraction Power" (*P*), given by expression (2) and the unit is expressed in diopters (D).(2)

*n*_*c *_(1.3375) is the corneal refractive index of the cornea often used for keratometric purposes [16].

## Methods

A target, which is the object projected onto the lachrymal film of the examined cornea, consists of several light spots (LEDs) displayed in a circle. The displacement, the size and deformation of the reflected image of these light spots are analysed providing the keratometry and the circularity of the suture.

The engineering of the system basically consists of a hand-held projection target; a video system for capturing the reflected image of the light spots from the cornea; a software for image processing and keratometric and circularity results.

### Hand-held projection target

**Figure 1:**
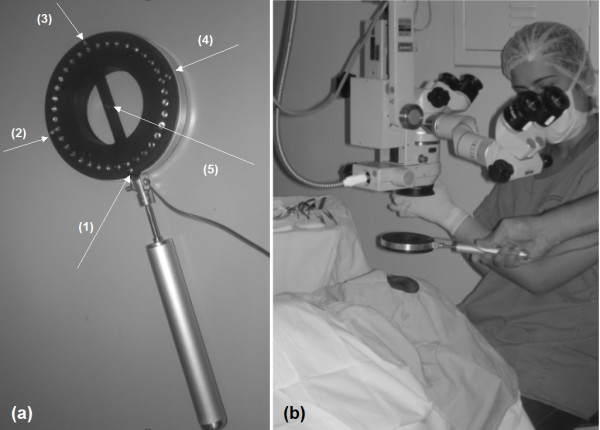
**(a) The projection system: Target with 36 LEDS displayed in a perfect circle to be used during a corneal suturing; (b) the target itself**.

The projection target has 36 infrared LEDs (high intensity - connected in parallel - 5 V and 50 mA) displayed in a perfect circle - ϕ = 53.8 mm -, 5 extra LEDS (one for center alignment of the system and 4 additional ones for angular alignment) - as shown in figure 1a - and which is to be held by the surgeon, as presented in figure 1b.

The target has a simple design that projects the light spots homogeneously in a precise circle of 3 mm of diameter in a standard cornea, i.e., with radius of curvature of 7.895 mm (a stainless steal sphere with precision of ± 0.0025 mm has been used as a standard cornea for calibrating the system). The light target should be held ideally 50 mm away from the cornea.

The handle of the luminous target is connected to a pedal that is placed under the surgical table. As the surgeon presses the left side, it lights up the luminous target; as the right side is pressed, the image is captured and the 5 guiding LEDs go off automatically. Alternatively, two push buttons may be placed at the handle for these purposes, but the surgeons who had been testing our system would rather have foot control.

As the target is turned on, it is focused by the surgeon at the examined eye. During the examination, the light from the surgical microscope is turned off.

**Figure 2:**
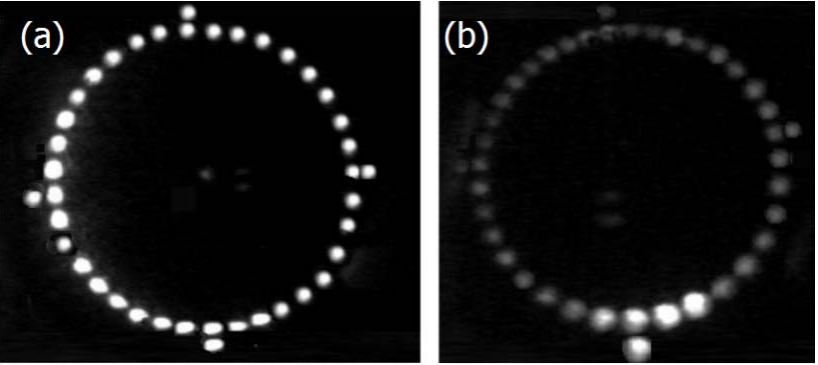
**Target projected onto: (a) a stainless steal sphere; (b) the patient's cornea during surgery**.

The image of the target projected onto the patient's cornea is reflected back through the central aperture of the target, straight forward to the objective of the microscope and then through the observation system. A beam-splitter is placed between the magnification system and the eyepieces of the microscope (70% of the reflected light is deviated to the eyepieces). A regular video optical adapter for surgical microscopes is coupled to the beam-splitter and a CCD camera WATEC 221S is attached to it. Figures 2a and 2b show the images of the target projected onto the calibrating sphere and onto the patient's cornea.

**Figure 3:**
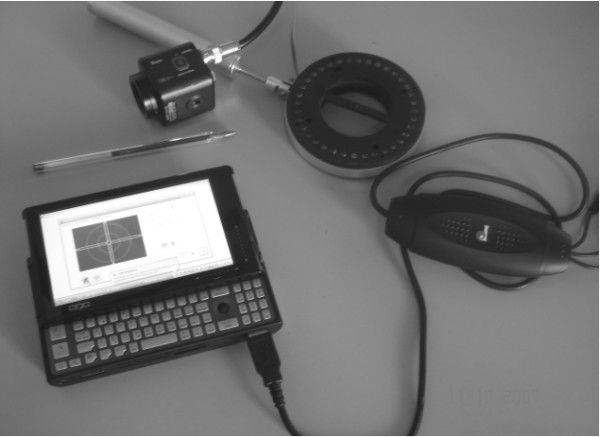
**Devices for assisting regular corneal suturing**.

The reflected image is analyzed by the software at a micro computer OQO - model 2 coupled to the CCD camera via a video bus cable (Pinnacle), as shown in figure 3.

While the surgeon is suturing the cornea, the physician should hold the target parallel to the cornea and align it guided by the 5 extra LEDs.

Manual focusing and image capturing processes take around 20-35 s depending on the familiarity of the surgeon with the prototype.

As the suture is being performed, the surgeon should instil some drops of saline and do the measurement. The image should be focused and the guiding LEDs 1-4 help to avoid tilting. The surgeon presses a pedal connected by the external cables, shown in figure 1b, that are connected to a micro computer by the USB port, and the image is captured. LEDs 1-5 are OFF at this time and just the remaining 36 LEDs are identified for keratometry.

The image is processed and the result is presented in a few milliseconds (typically 130 ms - refer to *processing time *in the software figure, furthermore).

The circularity of the cornea is presented as well as the keratometry, to avoid flattening or over bending of the cornea.

### The developed software

The developed software for supporting corneal suturing consists of 5 fundamental parts: image processing; keratometry measurement; circularity indication; user interface; calibration routine.

### Image Processing

The software is based on the information provided by the structure of the projecting target, determining its distortion as its image is reflected back from the cornea. The distortions analyzed between the original shape of the projected target and its reflected image contains information to provide the keratometric data.

Differently from the usual keratometers, the keratometric module for the ocular microscope is not usually operated in an environment free from external disturbances, as room light, for example. Hence, the developed algorithm is capable to avoid these kinds of interferences in the image processing.

Non-uniformity of brightness distribution in the image of the light spots that reach the examined surface results in loss of information in the identification process of the target's shape. The developed algorithm considers this possibility, once it provides the keratometric results even if some light spot is missing in this process.

There is a considerable variation of the brightness of the light spots, in the grayscale image. Therefore, a blurring process is used by a matrix 3 × 3 as:(3)

Subsequently, a convolution process is applied, using a circular mask with diameter based on the average diameter of the light spots, providing means to identify each LED position in the image.

As the mask is moved along the image, the pixel corresponding to the highest intensity is the first to be identified.

The center of mass having a well defined position is identified and its location is saved as (*Px*[1], *Py*[1]) Proceeding in a recursive way, the lower intensity structures are then identified

As the N_e _(LEDs) structures are identified, the positioning of their respective centre of mass (*Px *[*i*], *Py*), (*x*_*cm*0_, *y*_*cm*0_) may be identified, providing the location of the centre of the whole image given as:(4)

Therefore, an interpolated curve is obtained from the position of the centre of mass of the reflected image of the target.

### Software for Keratometric Measurements

**Figure 4:**
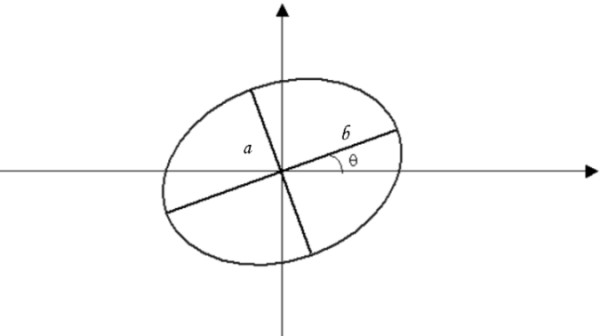
**Representation of the ellipse used in the software's algorithm**.

The mathematical fitting expression of the reflected image of the target onto the cornea is an ellipse, with an inclination angle of θ related to the x axis of the coordinate system, as represented in figure 4. The coordinate system is X, Y and *a *and *b *are the minor and major axis of the ellipse, respectively.

The fitting is performed using the interpolated data determined from the image processing.

These data were defined in polar coordinates for the angles Θ_*i *_= *n*_*i*_, Θ_0_, where 0° ≤ Θ_*i *_≤ 360°. In order to guarantee accuracy of the measurements, the angle was chosen to be Θ_0 _= 1° and *n*_*i *_= 0,1,2,3, ..., 359.

An ellipse with its centre coincident with the centre of mass of the image, as shown in figure 4 may be written as:(5)

The best fitting ellipse is determined from comparing it to a trial ellipse with an inclination angle of *θ *= *n*_*i *_Θ_0_, where 0° ≤ *θ *≤ 90°. For each value of *n*_*i*_, the mean square root is calculated among the interpolated points and the actual points from the determined ellipse of the interpolation of the median curve from the ring. The mean square deviation is given by:(6)

*r*_*i *_corresponds to the radius obtained from the fitted curve (as a function of Θ_*i*_) and *r *is the radius of the interpolation of the median curve from the ring, defined by the transformation (*Px *[i], *Py *[*i*]) → (r [i], Θ [*i*]), i.e., x = *r *cos(Θ_*i*_) and *y *= *r sen*(Θ_*i*_).

In order to determine the ellipse that best fits to the image, we have chosen the equation with the least attributed value of *S*. The conversion from pixels to millimeters is necessary in order to present the radius of curvature of the surface being measured.

### Eccentricity

The eccentricity is a function added to the software to acquaint the surgeon with the circularity of the suturing, indicating whether the surgeon should stiffen the stitch or relax it.

The eccentricity, denoted *e*, is a parameter associated with every conic section. It can be thought of as a measure of the deviation of the conic section from a circular shape [17]:(7)

For any ellipse, let *a *be the length of its semi-major axis, *b *be the length of its semi-minor axis and K = 1.

Therefore, the circularity has been defined as(8)

It should be noted that for the ellipse 0<e<1, therefore circularity varies from 0% - 100%.

### Data Screen

**Figure 5:**
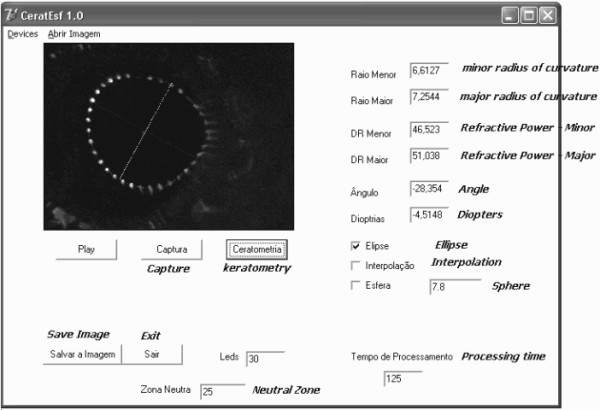
**One of the software's screens used for our own control - it is not presented to the surgeon**.

Figure 5 presents a picture of the software screen used for our own control and that is not presented to the surgeon.

It presents all the measurements that we should have access to, including the elapsing time of the captured image and the presented result, in another words the processing time - "tempo de processamento"; the type of approximation being performed (elliptical); the polynomial interpolation required if the number of identified LEDs is not enough; the radius of curvature of the calibration sphere; in this case 7.8 mm; the number of LEDs that could be identified - in this case 30; the neutral zone - "zona neutra", where LEDs are not supposed to be identified - in the center, for example; etc.

### The interface software and calibrating system

**Figure 6:**
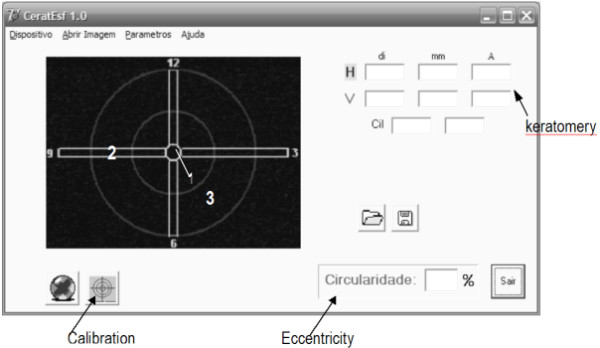
**Screen presented to the surgeon during suturing**.

A real time image is displayed at the OQO monitor with orientations for adjusting the target's axis and center. Figure 6 presents the interface screen. There is a center guide denoted by 1 (small circle at the center of the image screen), as well as an angular guide, denoted by 2 (two rectangles orthogonally placed in the center of the image capturing screen). Region denoted by 3 is the location that the 36 reflected spots should be restrained.

There is a calibration button for the system. The calibration of the system should be performed whenever its optical components are removed and then replaced back in the microscope.

It consists in positioning a standard stainless sphere with radius of curvature of 7,895 mm ± 0,0025 mm at a pre-determined proper location; set the microscope to a desired magnification and capture its reflected image from the 36 light spots.

Keratometry and circularity (eccentricity) are presented whenever the image is captured (either by the mouse, voice command, or stepping pedal). The item referring to devices "dispositivos" on the top bar of figure 6, the surgeon may choose one of these options.

**Figure 7:**
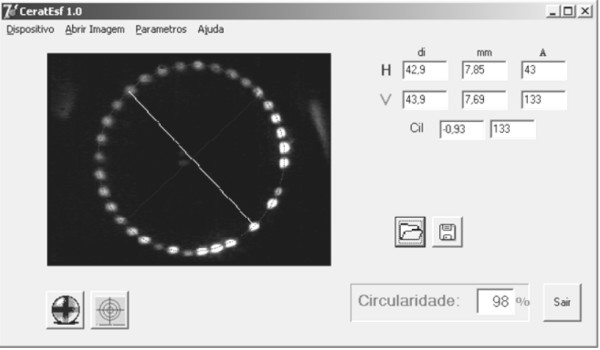
**Real time surgery measurement of keratometry and eccentricity of a cataract surgery**.

Figure 7 presents a measurement performed in a patient during suturing. The surgeon has achieved 98% of eccentricity, however, it shows that almost 1D of astigmatism remains.

Statement of ethical approval: This research has been approved by the Ethics Committee of the Clinical Hospital of Ribeirão Preto - CAAE (Certificate for Ethical Appreciation) - reference number: 0212.0.004.000-09.

## Results

The prototype has been submitted to standard tests, in order to check its reliability and its accuracy, prior to performing clinical trial on intra operative patients. Thus, it was first checked by using standard steal spheres; then its focal depth has been determined; followed by testing the prototype in post operative patients. The tests were performed to some extent to obtain data regarding the residual astigmatism that corneal transplanted patients usually present 24 hours after the surgery, as well as comparing the keratometric data of these patients with a hand held commercial keratometer, supplying their correlation factor; and finally using it intra operatively and checking the residual astigmatism within 24 hours after the surgery.

In order to know the accuracy of our system, 21 standard steal spheres have been analyzed. The correlation of our system and the data provided by the manufacturer of the standard spheres, in a range of 3.0000 mm - 17.0000 mm ± 0.0025 mm is 0.99.

Spheres with radius of curvature of 7.8 mm, which best represent the corneal model, is 90% reproducible, presenting an error of 3%. They are used for calibrating the system. For tested spheres with larger radius of curvature, the reproducibility is 95% and for smaller radius of curvature, it is 87%, due to critical centralization and alignment procedures.

In order to evaluate the accuracy on measuring a distorted surface, three contact lenses have been distorted in a wedge, placed on a device that could rotate the lenses precisely every 1° for all 360°, and have been used as trial surfaces. These lenses were measured in a Topcon OM-4 keratometer (three times each) and in our prototype. Table 1 presents the obtained data for lens 1.

**Table 1:** Obtained refractive power and astigmatism's assiociated axis for lens 1 in keratometer Topcon OM-4 and Prototype, and compared to theorethical data.

Rotation angle	Theoretical Expected Value	Measurements on Topcon OM-4		Measurements on Prototype	
		(D)	θ (degrees)	(D)	θ (degrees)
0	42,2500	41,70	0	42,62	0
1	42,2505	41,70	1	42,62	1
2	42,2518	41,70	2	42,62	2
3	42,2541	41,70	3	42,63	3
4	42,2573	41,71	4	42,63	4
5	42,2614	41,71	5	42,63	5
6	42,2664	41,72	6	42,64	6
7	42,2723	41,72	7	42,64	7
8	42,2791	41,73	8	42,65	7
9	42,2867	41,74	8	42,66	8
10	42,2953	41,75	9	42,67	9
11	42,3047	41,75	11	42,68	11
12	42,3149	41,76	12	42,69	12
13	42,3260	41,78	13	42,70	13
14	42,3378	41,79	14	42,71	14
15	42,3505	41,80	15	42,72	15
16	42,3640	41,81	16	42,74	16
17	42,3783	41,83	17	42,75	16
18	42,3933	41,84	18	42,77	17
19	42,4091	41,86	19	42,78	18
20	42,4255	41,87	20	42,80	19
21	42,4427	41,89	21	42,82	20
22	42,4606	41,91	22	42,83	21
23	42,4791	41,93	22	42,85	22
24	42,4982	41,95	23	42,87	23
25	42,5180	41,97	24	42,89	24
26	42,5383	41,99	25	42,91	25
27	42,5593	42,01	26	42,93	26
28	42,5807	42,03	26	42,96	27
29	42,6027	42,05	28	42,98	28
30	42,6251	42,07	29	43,00	29
31	42,6480	42,09	29	43,02	30
32	42,6713	42,12	30	43,05	31
33	42,6951	42,14	31	43,07	32
34	42,7191	42,16	33	43,10	33
35	42,7436	42,19	34	43,12	34
36	42,7683	42,21	35	43,14	35
37	42,7934	42,24	36	43,17	36
38	42,8187	42,26	37	43,20	37
39	42,8442	42,29	38	43,22	38
40	42,8699	42,31	39	43,25	39
41	42,8957	42,34	40	43,27	40
42	42,9217	42,36	41	43,30	41
43	42,9478	42,39	42	43,33	42
44	42,9739	42,42	43	43,35	43
45	43,0001	42,44	44	43,38	44
46	43,0263	42,47	45	43,40	45
47	43,0524	42,49	46	43,43	46
48	43,0785	42,52	47	43,46	47
49	43,1045	42,54	48	43,48	48
50	43,1303	42,57	49	43,51	49
51	43,1560	42,60	50	43,54	49
52	43,1815	42,62	51	43,56	50
53	43,2068	42,65	52	43,59	51
54	43,2319	42,67	53	43,61	52
55	43,2566	42,69	54	43,64	53
56	43,2810	42,72	55	43,66	54
57	43,3051	42,74	56	43,69	55
58	43,3289	42,77	57	43,71	56
59	43,3522	42,79	58	43,73	57
60	43,3751	42,81	59	43,76	58
61	43,3975	42,83	60	43,78	59
62	43,4195	42,86	61	43,80	60
63	43,4409	42,88	62	43,82	61
64	43,4618	42,90	63	43,84	62
65	43,4822	42,92	64	43,86	63
66	43,5019	42,94	65	43,88	64
67	43,5211	42,96	66	43,90	65
68	43,5396	42,97	67	43,92	66
69	43,5574	42,99	68	43,94	67
70	43,5746	43,01	69	43,96	68
71	43,5911	43,02	70	43,97	69
72	43,6068	43,04	71	43,99	70
73	43,6218	43,05	72	44,01	71
74	43,6361	43,07	73	44,02	72
75	43,6496	43,08	74	44,03	73
76	43,6622	43,09	74	44,05	74
77	43,6741	43,11	75	44,06	75
78	43,6852	43,12	76	44,07	76
79	43,6954	43,13	77	44,08	77
80	43,7048	43,14	78	44,09	78
81	43,7133	43,15	79	44,10	79
82	43,7210	43,15	80	44,11	80
83	43,7277	43,16	81	44,11	81
84	43,7336	43,17	82	44,12	81
85	43,7386	43,17	83	44,12	82
86	43,7427	43,17	84	44,13	83
87	43,7459	43,18	85	44,13	84
88	43,7482	43,18	86	44,13	85
89	43,7495	43,18	87	44,13	86
90	43,7500	43,18	88	44,13	87

The correlation factor between our system and Topcon OM-4 regarding the refractive power is r^2 ^= 0,94, and r^2 ^= 0,97 for the axis. Same procedure has been adopted for lenses 2 and 3 and the correlation factor was the same for these later ones.

### Focus Depth

Bonnet [18] stated that keratometers should have focus depth inferior to 0.35 mm, with the intention that the precision on the radius of curvature of the tested surface be 0.03 mm. The focus depth measurements were performed in our system using the 10× magnification (the most commonly used magnification for the surgeries at our service), by focusing the image of the target onto the precision sphere. The measuring procedure was performed 60 times. For every test the system was completely defocused and then the image was brought into focus by two different users (30 times each). The focus depth in our hand held system is 0.08 mm, leading to a ± 0.25D error.

### Keratometry Tests on Post Operative Patients and Circularity

In order to evaluate regular residual astigmatisms after a corneal transplantation and to understand the range of circularity that a surgeon may be working with, so that astigmatisms over 3D are not achieved, it is necessary to evaluate several eyes to have them as guiding data. Thus, 290 post operative patients, who were not submitted to the use of the prototype during surgery, had keratometry performed, as well as circularity measurements.

### Keratometry

Keratometry in postoperative patients (290), who had not yet been submitted to the surgery with the support device for suturing information, were performed in our system and in a Topcon OM-4 keratometer. These individuals have been examined to provide information of the immediate postoperative residual astigmatism, as well as to compare the effectiveness of measurements performed with the prototype with a commercial available keratometer. The patients were tested on both systems. The patients were divided into 2 groups: corneal transplants (78%); and cataract (32%). Astigmatisms greater than 8D were commonly reported. The data obtained for these astigmatisms vary from 0.75D - 11.85D. The average was 7.95D. Both systems were previously calibrated and the exams performed on both systems were 0.97 correlated.

These measurements were performed to assure that the system was in agreement with commercial available systems to perform accurate keratometry.

Also 72 of these patients were submitted to keratometry in the sitting up position and in the lying down position, and were evaluated by the developed system and the Nidek Oculus Hand-held corneal topographer, which allows measurement on a lying down position. The keratometry obtained for these patients differ from the lying down position to the sitting up position. Keratometric values were reported to show a discrepancy up to 1.5D in some cases.

The correlation factor between our system and the Nidek Oculus Hand-held corneal topographer for the sitting up position was r^2 ^= 0.97 and for the lying down position r^2 ^= 0.96.

Thus we have concluded that our prototype was able to perform keratometry in volunteer patients and the surgeons were aware that the most important parameter to be used in the software for suturing purposes was the circularity.

### Circularity

It has been registered for the 290 trial patients that in order to keep residual astigmatisms lesser than 3D for patients with corneas presenting radii of curvature around 7.8 mm, the minimum circularity that must be achieved is 97%; for flatter corneas (around 38D), the minimum circularity to be achieved is 95%; and for a steeper cornea, the minimum required circularity is 99%.

### Intraoperative Testing

Subsequently to the evaluation tests of the accuracy of the system itself, and knowing the expectation of pos operative residual astigmatism, the prototype was tested in 14 patients during corneal transplant surgery.

Patients have been submitted to our system during surgery for guiding suturing by the circularity data. Then, the patients were submitted to our prototype and the Nidek Oculus Hand-held corneal topographer, 24 hours after the surgery.

Table 2 presents the circularity measurements performed in 14 volunteer patients during surgery (corneal suturing) in the lying down position and the comparison of postoperative keratometric measurements performed on those patients in the sitting down position on the prototype and on the Nidek Oculus Hand-held corneal topographer.

**Table 2:** Circularity measurements during surgery and postoperative keratometric measurements performed in 14 volunteer patients

Patient	Nidek Oculus Hand-held corneal topographer Post-operative Remaining Astigmatism (diopter)	Prototype Post-operative Remaining Astigmatism (diopter)	Prototype - Achieved Circularity (%)	Prototype Axis (degrees)	Nidek Oculus Hand-held corneal topographer Axis (degrees)
1	-0,75	-0,75	99	24	5
2	-0,50	-0,75	98	2	1
3	-3,00	-2,25	92	1	2
4	0,00	-1,50	95	0	3
6	-1,00	-1,25	94	104	110
7	-1,50	-1,75	95	180	171
8	-4,50	-4,75	85	32	30
9	-0,75	-0,50	99	57	33
10	-0,50	-0,50	99	16	4
11	-1,25	-1,00	99	15	8
12	-0,50	-0,75	97	102	73
13	-0,25	-0,75	98	148	134
14	-2,50	-2,75	90	162	156

**Figure 8:**
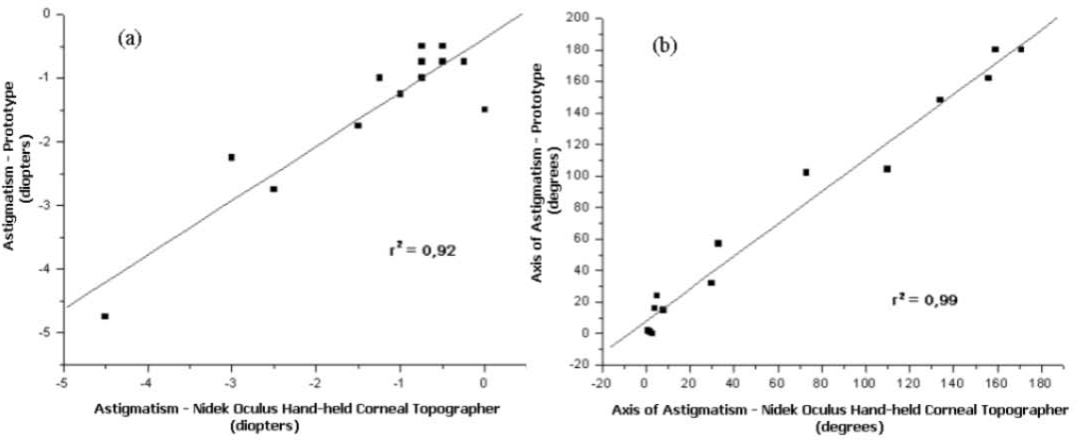
**Correlation factors between the postoperative measurements performed in patients (sitting down position) using the prototype and the Nidek Oculus Hand-held corneal topographer**.

The keratometry results have correlation factors of r^2 ^= 0.92 for the refractive power and r^2 ^= 0.99 for the axis, with the sutures still on the cornea. Figure 8 presents the correlation factors of the performed measurements.

## Discussion

The system is able to provide up to 23D of astigmatism (32D - 55D range), with accuracy of ± 0.25D.

Regarding the 14 tested patients, astigmatisms varying from 0.5D - 4.75D were obtained in the postoperative.

It can be noted that surgeons tend to tight the stitches, resulting in negative power astigmatisms.

The average of residual astigmatism for this relatively small number of patients was -1.4D, which leads to a positive contribution of the use of this device for suturing.

This average is significantly lesser than the average of 8.0D obtained for the patients that were not submitted to surgeries benefitted by the use of the prototype.

Considering figure 7, it may be noticed that high postoperative astigmatisms tend to be easily induced by the irregular manual suturing of the tissue. Even achieving a high eccentricity (98% of circularity) during the sutures, around 1D of astigmatism still remains.

It has been observed that circularity is a very intuitive parameter for the clinician to manage the stitches during surgery, but the surgeon should also be aware that it may be difficult to achieve circularity close to ideal (100%) depending on the curvature of the patient's cornea. For instance, in order to keep induced astigmatisms smaller then 3D, for patients having corneas with radii of curvature around 7.8 mm, a 97% of eccentricity should be achieved; for flatter corneas (refractive power of 38D) and more warped corneas (refractive power of 55D), eccentricities of 95% and 99%, respectively should be accomplished. Therefore, for steeper corneas, high circularity should be achieved for best postoperative results.

**Figure 9:**
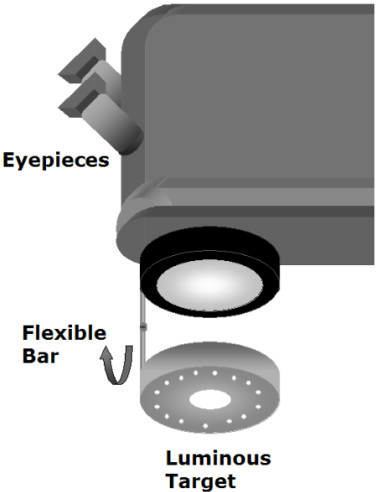
**Schematics of the suggested prototype for clinical routine use**. A flexible bar for a fixed target system is attached to the objective of the microscope.

One of the limitations of this present prototype for clinical routine is that it is not of easy focusing or aligning the target during surgery. In order to overcome this problem, we suggest that the target should be attached to the objective of the microscope, using a flexible rod, so that the target could be folded down for its use and then folded back up while not using it, as presented in a schematic drawing in figure 9.

## Conclusion

The obtained results encourage re-designing the prototype to turn it into a clinical routinely used prototype. Suggestions previously made (refer to figure 9) may be an easy solution for a more comfortable and free-hands use of the device. The system should be fixed at the microscope for a better ability in its use. Also focusing and centering becomes easier. Some of the surgeons have difficulty in aligning the system. However, we were not, at a first moment, concerned about using the instrument in a clinical routine, we were just enthusiastic on finding out if the orientation for suturing would be of any benefit for the patient, even at the very first post operative days (unstable astigmatism is quite frequent in this period), to provide better vision quality.

We have observed that there has been a significant reduction in the post-operative astigmatism when the system is used during surgery.

Follow ups for the next 24 months are still being performed, but we have ultimately provided for these patients a better immediate post-operative quality of vision.

Now, we are adapting the system in the microscope, to turn it into a routine at the hospital that it has been tested.

These preliminary results obtained for the 14 patients may lead to an awareness that manual suture should be carefully performed in order to obtain better postoperative results related to remaining astigmatism.

Perhaps automated systems for suturing should be considered in the near future.

## Competing interests

The authors declare that they have no competing interests.

## Authors' contributions

LV is the author of the concept idea and designed the optical parts and the electronics project and has given some contribution on the software implemented; JJG is responsible for actually programming the software, SJFeS is the medical doctor who has evaluated most of the patients in the prototype, PS has implemented the electronics. All authors read and approved the final manuscript.
